# Characterization of the nasopharyngeal and middle ear microbiota in gastroesophageal reflux-prone versus gastroesophageal reflux non-prone children

**DOI:** 10.1007/s10096-017-3178-2

**Published:** 2018-02-05

**Authors:** Stefan A. Boers, Marjolein de Zeeuw, Ruud Jansen, Marc P. van der Schroeff, Annemarie M. C. van Rossum, John P. Hays, Suzanne J. C. Verhaegh

**Affiliations:** 1 Department of Medical Microbiology and Infectious Diseases, Erasmus MC, University Medical Center, Rotterdam, The Netherlands; 2 Department of Molecular Biology, Regional Laboratory of Public Health, Haarlem, The Netherlands; 3 Department of Otorhinolaryngology, Erasmus MC-Sophia Children’s Hospital, University Medical Center, Rotterdam, The Netherlands; 4 Department of Pediatrics, Division of Pediatric Infectious Diseases and Immunology, Erasmus MC-Sophia Children’s Hospital, University Medical Center, Rotterdam, The Netherlands

## Abstract

Otitis media (OM) is one of the most common pediatric infections worldwide, but the complex microbiology associated with OM is poorly understood. Previous studies have shown an association between OM and gastroesophageal reflux (GER) in children. Therefore, in order to bridge the gap in our current understanding of the interaction between GER and OM, we investigated the nasopharyngeal and middle ear microbiota of children suffering from GER-associated OM and OM only, using culture-independent 16S rRNA gene sequencing. Middle ear fluid, nasopharyngeal swabs, and clinical data were collected as part of a prospective pilot study conducted at the Department of Otorhinolaryngology of the Erasmus MC-Sophia Children’s Hospital, Rotterdam, the Netherlands. A total of 30 children up to 12 years of age who suffered from recurrent acute otitis media (AOM) (5), chronic otitis media with effusion (OME) (23), or both (2), and who were listed for tympanostomy tube placement, were included in the study. Nine children were included in the GER-associated OM cohort and 21 in the OM-only cohort. We found no obvious effect of GER on the nasopharyngeal and middle ear microbiota between the two groups of children. However, our results highlight the need to assess the true role of *Alloiococcus* spp. and *Turicella* spp. in children presenting with a high prevalence of recurrent AOM and chronic OME.

## Introduction

Respiratory tract infections are the leading causes of morbidity and death in children and adults worldwide, with otitis media (OM) being one of the most prevalent pediatric respiratory tract infections [1]. OM causes severe pain and can lead to serious complications, such as meningitis, mastoiditis, and hearing loss. As an example, OM-related hearing impairment has a prevalence of 30.82 per 10,000, and, each year, 21,000 people die due to complications of OM (estimated from 21 World Health Organization [WHO] regional areas) [2]. Importantly, the pathogenesis of OM is multifactorial, involving genetic, microbiological, and environmental factors [3].

A potentially important, though relatively unknown, factor to be associated with OM is gastroesophageal reflux (GER). GER, also known as acid reflux, is a clinical manifestation in which the stomach’s contents return back up into the esophagus and mouth. Reflux is a normal process that occurs in healthy infants, children, and adults. Most episodes are brief and do not cause bothersome symptoms or complications. However, earlier studies have shown a strong association between GER and OM [4–6]. The mean prevalence of GER in children with (chronic) OM with effusion (OME) is 48% and in children with (recurrent) acute OM (AOM) 63%, while the mean prevalence of GER in healthy children is less than 10% [7]. The data suggest a role for GER in the multifactorial etiology of OM in children, a hypothesis strengthened by the detection of pepsin and bile acids in the middle ears of children presenting with OM [4–6], as well as the possible role of *Helicobacter pylori* in the etiology of OM [8–11]. However, to date, no one has used culture-independent techniques to investigate the possible influence of GER on the nasopharyngeal and middle ear microbiota in GER-prone children. In this respect, differences in the presence or absence of (specific) microorganisms in the middle ear of GER-prone versus GER non-prone children could help further establish a role for GER in the pathogenesis of OM.

Therefore, in order to bridge the gap in our current understanding of the interaction between GER and OM, we investigated the nasopharyngeal and middle ear microbiota of children suffering from GER-associated OM and OM only, using culture-independent 16S rRNA gene sequencing.

## Materials and methods

Middle ear fluid (MEF), nasopharyngeal swabs (NPS), and clinical data were collected according to Klokkenburg et al. [4] and Stol et al. [12], as part of a prospective pilot study conducted at the Department of Otorhinolaryngology of the Erasmus MC-Sophia Children’s Hospital, Rotterdam, the Netherlands. Children with a history of craniofacial malformations/syndromes, or primary or acquired immune deficiency were excluded from participation. Questionnaires were used to obtain relevant patient characteristics from the parents of the GER-associated OM cohort and the OM-only cohort. The GER-associated cohort was defined via questionnaires based on the questions “Has your child been diagnosed with reflux in the previous three months?” and “Has your child been diagnosed with reflux in the past?” The study was approved by the Erasmus MC Medical Ethical Committee (MEC-2012-487). Written informed consent was obtained from parents or caregivers.

Microbiota profiles were analyzed using next-generation sequencing of hypervariable V5-V6 regions of the 16S rRNA gene, as previously described [13]. Prior to 16S rRNA gene sequencing, the total number of 16S rRNA gene copies within each DNA extraction was measured using a 16S quantitative polymerase chain reaction (PCR) according to Yang et al. [14]. This was performed in order to remove any specimens from the analysis where the contaminating bacterial DNA that is already present within chemicals and consumables used in the experimental procedure could be responsible for the results obtained. In this respect, all specimens that contained < 1000 16S rRNA gene copies/μL were not sequenced [15]. All sequence data obtained from specimens containing > 1000 16S rRNA gene copies/μL was processed using bioinformatics modules present in the mothur v.1.33.0 software package [16].

Statistical analyses were performed using IBM SPSS Statistics 21. Fisher’s exact test was used to calculate the statistical differences between patient baseline characteristics, differences between antibiotic use and specimen exclusion, antibiotic use, and pathogen detection, and to calculate the statistical differences of prominent middle ear microbiota between GER-prone and GER non-prone children. A *p*-value of ≤ 0.05 was considered to be statistically significant.

### Data availability

The datasets generated and analyzed during the current study are available in the NCBI Sequence Read Archive (SRA) repository with the accession number SRP099862, https://www.ncbi.nlm.nih.gov/sra/?term=SRP099862.

## Results and discussion

### 16S rRNA gene sequencing

Thirty children up to 12 years of age who suffered from recurrent AOM (5), chronic OME (23), or both (2), and who were listed for tympanostomy tube placement, were included in the study (Table 1). Children were diagnosed based on the surgeon’s report.

**Table 1:** Demographic and clinical parameters stratified by the gastroesophageal reflux (GER)-associated otitis media (OM) cohort (*n* = 21) and the OM-only cohort (*n* = 9)

	No GER, *n* = 21 (%)	GER, *n* = 9 (%)	*p*-Value
Demographic parameters			
Male sex	13 (61.9)	5 (55.6)	1
Age, years	5.3 [1.3–6.0]	3.7 [0.8–12.8]	
Antibiotics ≤ 6 months preceding inclusion	12 (57.1)	5 (55.6)	1
Siblings	16 (76.2)	5 (55.6)	0.39
Day-care	7 (33.3)	7 (77.8)	0.05
Smoking	14 (66.7)	5 (55.6)	0.69
Breastfeeding	11 (52.4)	8 (88.9)	0.10
Diagnosis			
OME	16 (76.2)	7 (77.8)	1
rAOM + (OME)	5 (23.8)	2 (22.2)	1
Ventilation tube placement in past	11 (52.4)	1 (11.1)	0.05
Adenotonsillectomy in past	7 (33.3)	0 (0)	0.07

In total, 11/30 MEF specimens and 11/30 NPS specimens (not, by definition, paired specimens) were excluded from 16S rRNA gene sequencing due to low DNA content (< 1000 16S rRNA gene copies/μL). Antibiotic use ≤ 6 months prior to inclusion in the study did not affect the exclusion of samples due to low DNA content (Table 2; MEF *p* = 0.13, NPS *p* = 0.45). The microbial composition of the remaining 38 specimens were sequenced and the data were processed using mothur. To reduce the effects of uneven sequencing depths, sequences were rarefied to 1000 sequences per specimen, which was sufficient to obtain a high degree of sequence coverage with an average Good’s coverage value of 0.98 (± 0.01).

**Table 2:** Inclusion and exclusion of samples related to antibiotic use ≤ 6 months preceding inclusion

	MEF		*p*-Value	NPS		*p*-Value
	Inclusion	Exclusion		Inclusion	Exclusion	
Antibiotics	13	4	0.13	12	5	0.45
No antibiotics	6	7		7	6	

Sequencing of the MEF microbiota resulted in the identification of 29 bacterial operational taxonomic units (OTUs) in 19 MEF specimens with an abundance > 1%, with an average of 4 OTUs (± 3) per specimen. Sequencing the nasopharyngeal microbiota identified 39 bacterial OTUs in 19 NPS specimens with > 1% abundance, with an average of 9 OTUs (± 4) per specimen.

### Characterization of middle ear bacterial communities

The most commonly detected OTUs were *Alloiococcus* spp. and *Turicella* spp., present in 12 and 11 MEF specimens, respectively. There has been some debate as to whether members of the bacterial genera *Alloiococcus* and *Turicella* actually play a role in the pathogenesis of OM in children or if they are part of the commensal microbiota (and invading the middle ear following perforation) [17–23]. Though *A. otitidis* and *T. otitidis* are frequently detected in OM children, it is questionable whether these organisms have enough pathogenic potential to induce OM, although studies have shown that *A. otitidis* may have enough immunogenic potential to modulate a host immune response [24–26]. In addition, *A. otitidis* is able to form both single- and multispecies biofilms with *Haemophilus influenzae*. When present in polymicrobial biofilms, *A. otitidis* can promote *H. influenzae* growth and survival by increasing biofilm production in adverse growth conditions and by altering antimicrobial resistance [27]. In this study, the use of antibiotics ≤ 6 months prior to inclusion in the study did not have an effect on the detection of *Alloiococcus* spp., *Haemophilus* spp., *Streptococcus* spp., or *Moraxella* spp. (Table 3). These findings are clinically important, as antibiotics overuse or misuse can promote the development of bacterial resistance to antibiotics. *Alloiococcus* spp. and *Turicella* spp. were not detected by 16S rRNA gene sequencing in any nasopharyngeal swab. Previous PCR- and culture-based studies from other populations have reported *A. otitidis* in 7–12% of nasopharyngeal swabs, in contrast to the study of Marsh et al., who found no *A. otitidis* in paired nasopharyngeal swabs [17, 18, 28, 29]. *Turicella otitidis* is isolated almost exclusively from middle ear exudates [20, 23]. However, our study was limited to 30 children and it is possible that *Alloiococcus* spp. and *Turicella* spp. would have been detected in a larger cohort of children. Failure to detect *Alloiococcus* spp. and *Turicella* spp. in nasopharyngeal swabs from any of the children with positive ear MEF samples suggests that they are unlikely to be primary otopathogens in this population, since nasopharyngeal colonization is considered to be the antecedent of OM [30]. To better understand the potential role of *Alloiococcus* spp. and *Turicella* spp. in OM, further studies into host interactions and colonization of adjacent sites in healthy and diseased children will be necessary. Ultimately, the findings of this study will need to be further validated in the future with larger cohort sizes.

**Table 3:** Antibiotic use and pathogen detection in middle ear fluid (MEF)

Pathogen	No. of specimens					
	GER (*n* = 6)		*p*-Value	GER (*n* = 13)		*p*-Value
	Without antibiotics (*n* = 2)	With antibiotics (*n* = 4)		Without antibiotics (*n* = 4)	With antibiotics (*n* = 9)	
*Alloiococcus* spp.	2	0	0.07	3	7	1
*Streptococcus* spp.	0	1	1	1	4	1
*Haemophilus* spp.	1	1	1	1	6	0.27
*Moraxella* spp.	0	0	1	0	1	1

**Fig. 1:**
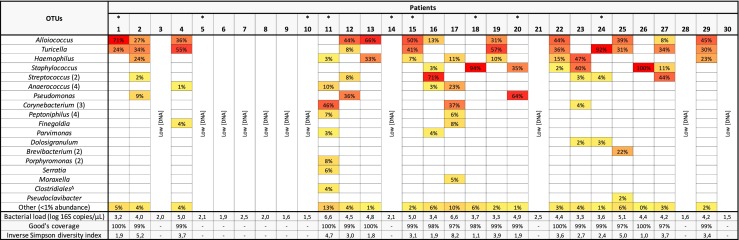
Heatmap demonstrating the relative abundance of bacterial taxa in individual middle ear fluid (MEF) specimens. Bacterial operational taxonomic units (OTUs) are grouped and shown at the genus level, unless the OTU was not identified below the taxonomic order level (^Δ^). A cut-off of 1% abundance was used for visual differentiation between specimens. The number of merged OTUs is shown in parentheses. Children that suffered from gastroesophageal reflux (GER) are indicated with an asterisk

OTUs consistent with classical otopathogens such as *Haemophilus* spp., *Streptococcus* spp., and *Moraxella* spp. were detected in 9, 6, and 1 MEF specimens, respectively (Fig. 1). In addition, staphylococci were also found in the MEF of seven children, with an abundance up to 100%. Our findings are consistent with several other studies; for example, *Haemophilus* (*influenzae*) has been found to be the most common pathogen isolated in the context of widespread conjugate pneumococcal vaccination [31, 32]. Further, the presence of *Haemophilus* (*influenzae*) has been associated with an increased risk of OM, whereas the presence of *Corynebacterium* spp. and *Dolosigranulum* spp. have been associated with a decreased risk of OM [33, 34].

No upper gastrointestinal tract microbiota previously associated with OM (e.g., *H. pylori*) was detected in the middle ear. Further, we did not find any microbial difference in the MEF of children with or without GER (Table 4), suggesting that GER was not associated with the translocation of stomach microbiota to the middle ear in our cohort.

**Table 4:** Effect of GER on the presence of prominent middle ear microbiota

Pathogen	No. of specimens		
	GER (*n* = 6)	No GER (*n* = 13)	*p*-Value
*Alloiococcus* spp.	2	10	0.13
*Haemophilus* spp.	2	7	0.66
*Streptococcus* spp.	1	5	0.60
*Moraxella* spp.	0	1	1

### Co-occurrence of OM-related pathogens

**Fig. 2:**
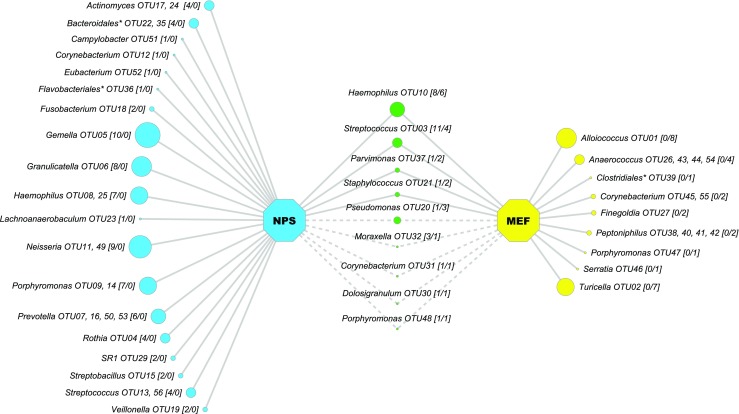
Co-occurrence model showing bacterial taxa present within 12 paired nasopharyngeal (NPS) and middle ear fluid (MEF) specimens. Data (Cytoscape v3.2.1) [37] are presented for 12 children, and include a single NPS and MEF specimen per patient. Taxonomic assignment for each OTU is at the genus level, unless the OTU was not identified below the taxonomic order level (*). A cut-off of 1% abundance was used for visual differentiation between both specimen types. Bracketed numbers [NPS/MEF] and node sizes indicate the number of specimens that contain a specified OTU. Shared OTUs are visualized in the middle and connected by a line if the presence of an OTU in both specimen types was derived from the same patient (solid) or from different children (dashed). Specimen-specific OTUs are grouped at the genus or order levels to ease visualization

To study the co-occurrence of OM-related pathogens in the nasopharynx and middle ear, we compared the microbiota obtained from 12 paired NPS and MEF specimens (Fig. 2). Nine OTUs were present in both specimen types, of which four OTUs, including *Haemophilus* spp., *Streptococcus* spp., *Parvimonas* spp., and *Staphylococcus* spp. were present in both paired specimen types from 10/12 children. Importantly, *Haemophilus* spp. and *Streptococcus* spp. were only detected in MEF when the nasopharynx of the child was concurrently colonized with these bacterial genera. This finding is in agreement with previous studies, and suggests that the more easily accessible nasopharyngeal microbiota could be used to predict the presence of pediatric otopathogens in the middle ears of children suffering from OM. However, it should be noted that the majority of OTUs, including the dominant genera *Alloiococcus* spp. and *Turicella* spp., were unique to the sampling site and, therefore, caution is necessary when using the nasopharyngeal microbiota as a proxy for the MEF microbiota, as discussed previously by van Dongen et al. [35].

### Strengths and limitations of the study

The strength of this publication is that it is the first study to investigate the possible association between the microbial composition of the nasopharynx and middle ear in GER-prone and GER non-prone children using culture-free 16S rRNA gene sequencing. However, several limitations in the study protocol should be recognized.

Firstly, an actual diagnosis of GER is difficult to prove, as most cases of pediatric GER are diagnosed based solely on the clinical presentation and parental observation (and not, for example, on the presence of pepsin and bile acids in the middle ear). There are no recognized classic physical signs of GER in the pediatric population, and, in general, a doctor diagnoses GER by reviewing an infant’s symptoms and medical history. In this study, only parental-reported clinical information was collected. Essentially, we asked parents retrospectively if their child was diagnosed with reflux in the previous three months and if their child had been diagnosed with reflux at any time in the past. To date, the most thoroughly evaluated questionnaire for infant symptoms is the Infant Gastroesophageal Reflux Questionnaire Revised (I-GERQ-R) [36]. We designed our own questionnaire, as even an established questionnaire such as the I-GERQ-R was not validated to be adequately specific to differentiate GER infants from other symptomatic, but non-GER, infants, particularly if the treatments being tested are directed at acid reflux. For that purpose, additional inclusion criteria, such as esophageal pH monitoring or histology, are currently necessary to ensure adequate diagnostic specificity [36]. In the current study, we did not confirm the parental-reported diagnosis of GER by the identification of bile acids/pepsin in MEF or measurement of the MEF pH levels. These tools have proven to be useful for determining the presence of pathological GER [4–6]. The absence of these tools in our study may have influenced the fact that we found no statistically significant association between the microbiota profiles of GER-prone and GER non-prone children.

Secondly, our study included only children primarily diagnosed with OME, as these children are mainly listed for tympanostomy tube placement. Most children with AOM experience a self-limiting illness and lack the presence of fluid in the middle ear. Many will not present to a doctor. This means that an association between GER and AOM disease is much more difficult to prove than between GER and OME disease, but does not affect the hypothesis that GER may be associated with increased OM disease in GER-prone children.

Thirdly, as previously discussed, using the more easily accessible nasopharyngeal microbiota to predict the presence of pediatric otopathogens in the middle ears of children suffering from OME and AOM is questionable. Because of the size of the population studied and lack of data from other studies, data interpretation should be made with caution and regarded as preliminary. Further, we were unable to analyze the microbiota to the species level (a common problem with microbiota analysis [13]) and were, therefore, limited to bacterial taxonomic identification at the genus level.
